# Mutational spectrum of hepatitis C virus in patients with chronic hepatitis C determined by single molecule real-time sequencing

**DOI:** 10.1038/s41598-022-11151-6

**Published:** 2022-04-30

**Authors:** Fumiyasu Nakamura, Haruhiko Takeda, Yoshihide Ueda, Atsushi Takai, Ken Takahashi, Yuji Eso, Soichi Arasawa, Eriko Iguchi, Takahiro Shimizu, Masako Mishima, Ken Kumagai, Taiki Yamashita, Shinji Uemoto, Nobuyuki Kato, Hiroyuki Marusawa, Akihiro Sekine, Hiroshi Seno

**Affiliations:** 1 Department of Gastroenterology and Hepatology, Graduate School of Medicine, Kyoto University, Kyoto, Japan; 2 Division of Gastroenterology, Department of Internal Medicine, Kobe University Graduate School of Medicine, Kobe, Japan; 3 Center of Preventive Medical Sciences, Chiba University, Chiba, Japan; 4 Department of Surgery, Graduate School of Medicine, Kyoto University, Kyoto, Japan; 5 Department of Tumor Virology, Okayama University Graduate School of Medicine, Dentistry, and Pharmaceutical Sciences, Okayama, Japan

**Keywords:** Viral genetics, Hepatitis C

## Abstract

The emergence of hepatitis C virus (HCV) with resistance-associated substitution (RAS), produced by mutations in the HCV genome, is a major problem in direct acting antivirals (DAA) treatment. This study aimed to clarify the mutational spectrum in HCV-RNA and the substitution pattern for the emergence of RASs in patients with chronic HCV infection. HCV-RNA from two HCV replicon cell lines and the serum HCV-RNA of four non-liver transplant and four post-liver transplant patients with unsuccessful DAA treatment were analyzed using high-accuracy single-molecule real-time long-read sequencing. Transition substitutions, especially A>G and U>C, occurred prominently under DAAs in both non-transplant and post-transplant patients, with a mutational bias identical to that occurring in HCV replicon cell lines during 10-year culturing. These mutational biases were reproduced in natural courses after DAA treatment. RASs emerged via both transition and transversion substitutions. NS3-D168 and NS5A-L31 RASs resulted from transversion mutations, while NS5A-Y93 RASs was caused by transition substitutions. The fidelity of the RNA-dependent RNA polymerase, HCV-NS5B, produces mutational bias in the HCV genome, characterized by dominant transition mutations, notably A>G and U>C substitutions. However, RASs are acquired by both transition and transversion substitutions, and the RASs-positive HCV clones are selected and proliferated under DAA treatment pressure.

## Introduction

Hepatitis C virus (HCV) is a positive-sense single-stranded RNA virus of the family of Flaviviridae. HCV became easily treatable through the use of direct acting antivirals (DAAs)^1^. However, emergence of HCV with resistance-associated substitutions (RASs) is a major concern for non-sustained virologic responses (SVRs) after DAA treatment^2–4^. Notably, the emergence of HCV with potent and/or multiple RASs after DAA treatment has made it difficult to eradicate HCV using DAA treatment^5–7^. However, the mechanism of evolution of HCV with potent and/or multiple RAS during DAA therapy has yet to be fully clarified.

Advances in sequencing technology have changed our understanding of the landscape of a HCV infection, which shows an abundant diversity and complexity in patients with chronic HCV infection^8^. Single molecule real-time (SMRT) sequencing, also known as third-generation sequencing, can generate extremely long contiguous sequence reads, while next-generation sequencing (NGS) reads short lengths (~ 400 bp)^8–11^. The long-read sequencing ability of SMRT sequencers makes it possible to determine the haplotype of individual HCV clones and clarify the linkage of RASs, which are far apart from each other in the same viral clone distributed from NS3 to NS5B. Moreover, the PacBio sequencer (Pacific Biosciences) has an extremely high accuracy conferred by circular consensus sequencing (CCS) methods, which can sequence a single template multiple times by creating a closed circle loop of the template^12,13^. In a previous study, we demonstrated that reading an identical template 10 times (i.e. 10-pass CCS reads) reduces the error rate to as low as 0.03% in the HCV genome^14^. By using long contiguous sequences spanning the NS3 to NS5A regions, we examined the origin of multi-drug resistant HCV clones emerged after DAA treatment. We also found that haplotypes and structural variations of the HCV genome occurred dynamically during treatment, according to the results of SMRT sequencing^15^.

The RASs in the HCV genome are thought to be produced as a result of spontaneous mutations of the HCV RNA, and some HCV with RASs can be selected in response to the selective pressure of DAAs^16,17^. However, the mechanism underlying the spontaneous mutations of HCV is not yet fully understood. HCV is a rapidly mutating virus, with a mutation rate ranging from 3.5 × 10^–5^ to 1.2 × 10^–4^ base substitutions/site/year^18–20^. These spontaneous rapid mutations result in a high genetic diversity, resulting in so-called “quasispecies”. The mutations in viral genomes are affected by multiple factors, including polymerase fidelity, sequence context, template secondary structure, cellular microenvironment, and replication mechanisms^21^. In the HCV genome, Powdrill et al. found that the HCV RNA-dependent RNA polymerase (RdRp) NS5B had a strong mutational bias in favor of transitions (i.e. A> , G>A, U>C, and C>U) substitutions over transversions^22^. Recently, Geller et al. clarified that the main factors responsible for the heterogeneity of HCV were base composition, the presence of high- and low-mutation clusters, and transition/transversion biases^20^. In fact, the HCV mutational repertoire was found to be dominated by transitions in comparison to transversions^20,22–24^. For example, using HCV replicon cell lines with long-term culture for 9 years, Kato et al. reported that transition mutations were more frequent than transversion mutations, resulting in an increase in the GC content of replicon RNA in a time-dependent manner^23^. However, the actual substitution changes of the HCV genome over time in a patient is difficult to identify by deep sequencing using next-generation sequencing due to the quasispecies nature of the HCV genome. Moreover, the substitution patterns would vary among patients depending on their situation (i.e. in natural course of chronic hepatitis C, during DAA treatment, or during interferon-based treatment).

In this study, we analyzed the features of mutations in the HCV genome in time courses of patients with or without liver transplantation as well as HCV replicons, using high-accuracy long contiguous SMRT sequencing. Taking advantage of this novel long-read sequencing technology, we examined the linkage of RASs of the HCV genome at single viral clone resolution, and phylogenetically investigated the mutational spectrum of HCV over time.

## Results

### Mutational spectrum in HCV replicon cell lines during 10 years’ culture

**Figure 1:**
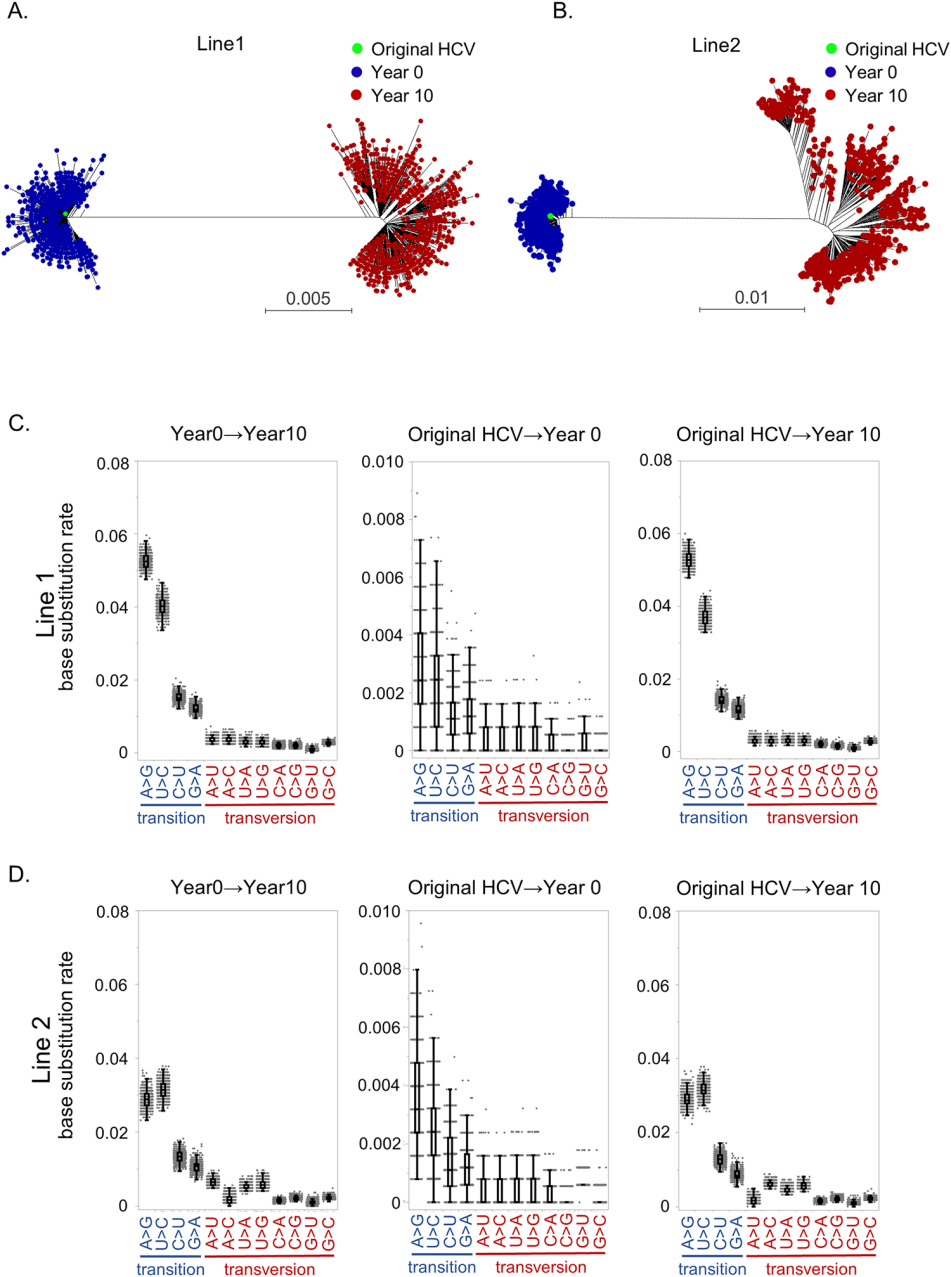
Phylogenetic analyses and mutational spectra in two HCV replicon cell lines during 10 years’ culture. (**A**, **B**) Phylogenetic analyses in HCV replicon cell line 1 (**A**) and 2 (**B**) during 10 years’ culture. Green dots denote the original HCV clones, which were originally transfected in the cell lines. Blue dots denote a subset of HCV clones at year 0, when the replicon cell lines established. Red dots demonstrate a subset of HCV clones at year 10 after 10 years’ culture from establishment. (**C**, **D**) Mutational spectra occurred during 10 years in the HCV replicon cell line 1 (**C**) and 2 (**D**) from year 0 to year 10 (left panels), compared between original clone and clones at year 0 (middle panels), and between original clone and clones at year 10 (right panels).

We first analyzed the spontaneous mutational spectrums in two HCV replicon cell lines, which were independently established and cultured for 10 years^23,25^. RNA extracted from these two cell lines at two different points (at year 0 when the replicon cell lines established and at year 10 after 10 years’ culture from the establishment) were amplified using HCV-specific primers and sequenced using the SMRT sequencing platform. The landscape of the HCV population was investigated by constructing phylogenetic trees from about 500 viral isolate sequences with 5- or more pass CCS reads to analyze the changes from year 0 to year 10 in two replicon cell lines (Table 1). Phylogenetic tree analysis demonstrated that the HCV clones at year 0 were widely distributed from the original HCV sequences transfected in the cell lines, indicating that many mutations occurred during the establishment of replicon cell lines (Fig. 1A,B). Surprisingly, phylogenetic analysis using HCV sequence of year 0 and year 10 revealed that the different clusters far away from the clusters at year 0 were generated at year 10 in both cell lines. This indicated the 10 years’ replication in HCV replicon cell lines resulted in many mutations in the HCV genome, selected a part of the HCV clones, and expanded from the selected clones (Fig. 1A,B). The number of mutations in the HCV genome that occurred from original clusters at year 0 to the clusters at year 10 were 178 in line 1 and 140 in line 2. In these mutations, 73 and 50 nonsynonymous mutations were identified from the NS3 to NS5B regions of the HCV genome in cell line 1 and 2, respectively (Supplementary Table 1). In the nonsynonymous mutations, amino acid changes at NS4A I29V, NS5A K378E, F380V/S, D402G/V, and L419P occurred at common amino acid positions in the two cell lines. It is worth noting that no known RASs for DAAs were contained in these mutations.

**Table 1:** Summary of the sequencing reads from HCV samples.

	Number of ≥ pass5 CCS reads	Number of CCS reads for phylogenic tree analysis	Read length analyzed (bps)
**Replicon Line1**			
Replicon (0 year)	16,229	733	5,972
Replicon (10 years)	18,858	722	5,972
**Replicon Line2**			
Replicon (0 year)	18,830	700	5,984
Replicon (10 years)	21,331	652	5,984
**Non-LT #1**			
Pre DCV/ASV	21,375	197	3,130
Post DCV/ASV	20,374	305	3,130
**Non-LT #2**			
Pre DCV/ASV	17,150	375	3,129
Post DCV/ASV	361	216	3,129
**Non-LT #3**			
pre DCV/ASV	12,138	508	3,028
post DCV/ASV	15,078	541	3,028
**Non-LT #4**			
Pre DCV/ASV	3578	216	3,075
Post DCV/ASV	19,719	228	3,075
**LT #1**			
Pre SMV	11,321	522	3,185
Post SMV	7729	539	3,185
Pre DCV/ASV	11,679	504	3,185
Post DCV/ASV	14,114	551	3,185
18M after DCV/ASV	17,197	505	3,185
**LT #2**			
Pre SMV	9369	642	3,261
Post SMV	10,210	634	3,261
Pre DCV/ASV	9028	652	3,261
Post DCV/ASV	13,033	642	3,261
18M after DCV/ASV	16,742	583	3,261
**LT #3**			
Pre SMV	5456	540	3,261
Pre DCV/ASV	7898	494	3,261
Post DCV/ASV	9595	483	3,261
18M after DCV/ASV	10,428	539	3,261
**LT #4**			
Pre SMV	12,116	596	3,261
Post SMV	14,696	573	3,261
Pre DCV/ASV	13,974	530	3,261
Post DCV/ASV	16,932	586	3,261
18M after DCV/ASV	13,870	561	3,261

*CCS* circular consensus sequencing.

By using these sequencing data, we analyzed the mutational spectrum during 10 years in the HCV replicon system. In the 12 possible base substitutions, transition substitutions (i.e. purine (A/G) to purine, or pyrimidine (C/U) to pyrimidine substitutions) were significantly more frequent than transversion substitution in the two HCV replicon cell lines (Fig. 1C,D). Moreover, in four transition substitutions, A>G and U>C transitions were significantly more frequent than C>U and G>A. These mutational biases were reproduced when we compared the original clone to clones at year 0 and clones at year 10 in both cell lines (Fig. 1C,D). These results clarified that this specific mutational spectrum occurred during the establishment of cell lines, and was maintained by selective pressure in the cell cultures over the long-term.

### Evolution of HCV diversity in patients with chronic HCV infection

We analyzed the dynamics of the HCV sequences in eight patients who received DAA therapy and did not achieve SVR, including four patients in non-LT settings and four patients after liver transplantation. The clinical characteristics of these patients are shown in Table 2. The results of the phylogenetic analysis after the SMRT sequencing of the four non-LT patients were reported as per our previous paper^14^. In addition, we analyzed four transplant recipients with serial anti-HCV treatments, interferon-based therapy with DAA, and interferon-free DAA therapy who did not achieve SVR.

**Table 2:** Clinical characteristics of non-LT and LT cases receiving DAA therapy.

Case	Age	Sex	LC or CH	HCV RNA (pre) (LogIU/ml)	Tacrolimus blood levels (ng/ml)	IL28B genotype	History of HCC	Previous DAA exposure	Reason for non-SVR
Non-LT #1	75	F	LC	6.6	–	NA	No	–	AE
Non-LT #2	61	F	CH	6.6	–	NA	No	TRV	VBT
Non-LT #3	63	F	LC	5.5	–	NA	No	SMV	AE
Non-LT #4	78	M	CH	5.4	–	NA	No	–	VBT
LT #1	63	M	CH	7.8	3.9	TT	No	SMV	NR
LT #2	63	M	CH	6.8	4.9	TG	No	SMV	VBT
LT #3	64	M	CH	6.9	9.1	TT	No	SMV	NR
LT #4	68	M	CH	7.6	6	TT	No	SMV	NR

*LT* liver transplantation, *DAA* direct acting antivirals, *M* male, *F* female, *LC* liver cirrhosis, *CH* chronic hepatitis, *SMV* simeprevir, *TRV* telaprevir, *SVR* sustained virological response, *VBT* viral breakthrough, *NR* non-responder, *AE* adverse event, – not detectable, *NA* not available.

**Figure 2:**
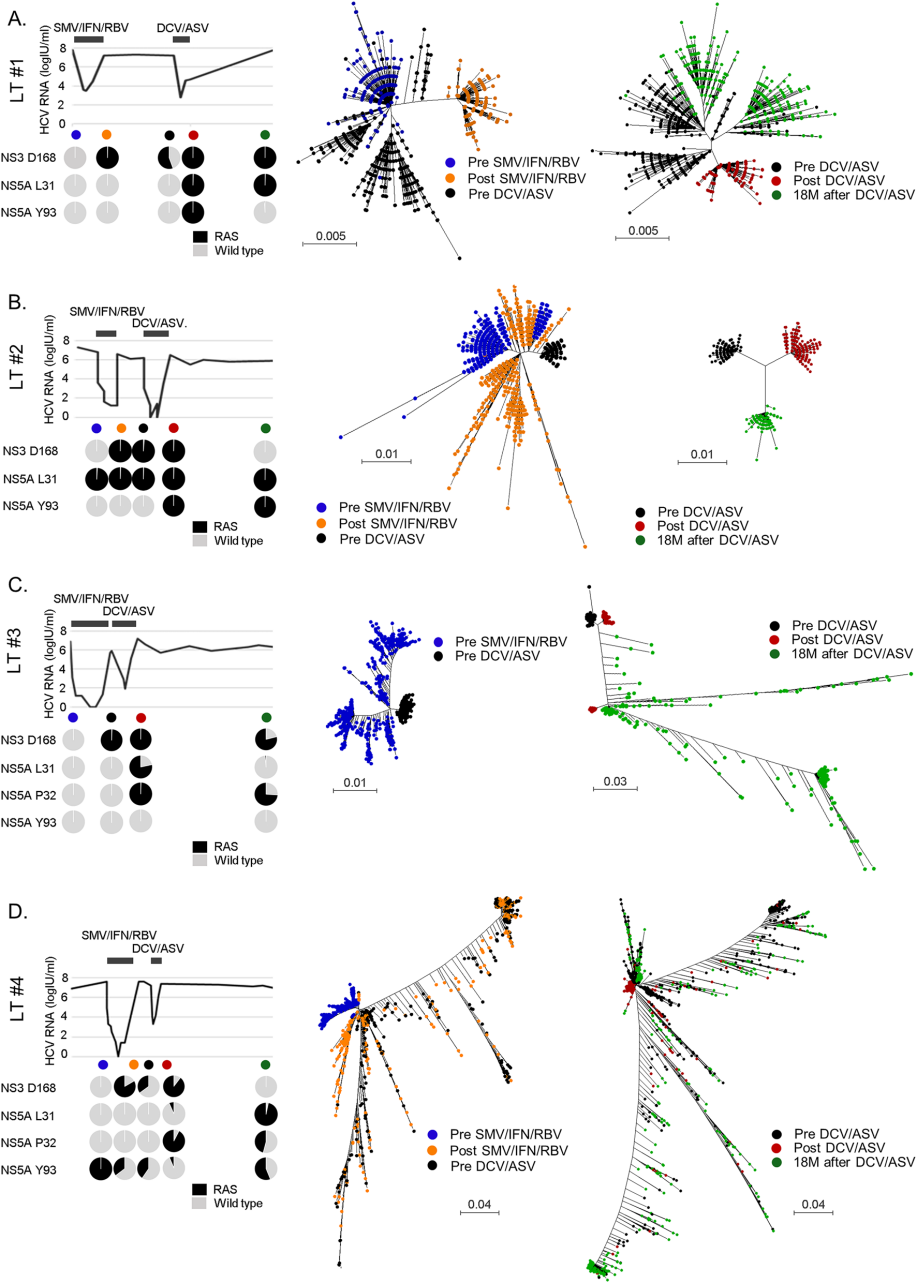
Clinical courses and phylogenetic analyses of four post-liver transplant (LT) patients during serial anti-HCV treatments by interferon-based therapy with DAA and interferon-free DAA therapy. Change of serum HCV RNA levels and HCV clones with resistance-associated substitutions at NS3 D168, NS5A L31, L32, and Y93 in four LT patients (left panels). Phylogenetic analyses of four LT patients at pre-simeprevir, interferon, and ribavirin therapy (SMV/IFN/RBV) (blue dots), post- SMV/IFN/RBV (orange dots), and pre- daclatasvir, and asunaprevir therapy (DCV/ASV) (black dots) (middle panels). Phylogenetic analyses of four LT patients at pre-DCV/ASV (black dots), post-DCV/ASV (red dots), and 18 months (M) post-DCV/ASV (green dots) (right panels).

The dynamic changes of the HCV clones with RASs analyzed by SMRT sequencing in the eight patients are shown in Table 3. Moreover, the changes in the serum HCV RNA levels and HCV clones with RASs at NS3 D168, NS5A L31, P32, and Y93 in the four LT patients are shown in Fig. 2A–D. HCV in all eight patients acquired multiple RASs after DAA treatment at NS3 D168, NS3 Q80, NS5A L31, NS5A P32, NS5A Q54, and/or NS5A Y93 (Table 3). By the linkage analysis of multiple RASs in each long viral sequence, most NS5A P32 deletion were detected exclusive with Y93H on the identical clones in LT patients #4 after daclatasvir and asunaprevir (DCV/ASV) treatment. Although a few minor clones have both P32 deletion and Y93H, they did not show any clonal expansion among the viral population (Supplementary Fig. 1).

**Table 3:** Population of HCV clones with resistance-associated substitution (RAS).

Case	Time point	The proportion of HCV clone with RAS						
		NS3		NS5A				
		D168V/M/A/E	Q80R/K	R30Q/K	L31M/V/F/I	P32del	Q54H/L	Y93H
Non-LT #1	Pre DCV/ASV	–	–	–	99.4	–	99.5	–
	Post DCV/ASV	88.9	–	–	99.5	–	99.2	99.9
Non-LT #2	Pre DCV/ASV	–	–	–	–	–	–	21.5
	Post DCV/ASV	100	–	–	99.7	–	–	100
Non-LT #3	Pre DCV/ASV	93.2	95.5	–	–	–	–	–
	Post DCV/ASV	99.5	99.6	–	99.2	–	99.2	99.9
Non-LT #4	Pre DCV/ASV	99.3	–	–	5.3	–	–	2.2
	Post DCV/ASV	98.2	99.8	99.5	99.7	–	–	99.8
LT #1	Pre SMV/IFN/RBV	–	–	–	–	–	–	–
	Post SMV/IFN/RBV	99.4	–	–	–	–	–	–
	Pre DCV/ASV	55.5	–	–	–	–	–	–
	Post DCV/ASV	99.9	–	–	99.4	–	–	99.9
	18 M after DCV/ASV	99.9	–	26.3	99.5	–	–	1.0
LT #2	Pre SMV/IFN/RBV	–	–	–	99.0	–	–	–
	Post SMV/IFN/RBV	99.7	–	–	99.3	–	–	–
	Pre DCV/ASV	98.7	–	–	99.5	–	–	–
	Post DCV/ASV	99.4	–	–	99.4	–	–	99.9
	18 M after DCV/ASV	–	–	–	99.7	–	–	99.7
LT #3	Pre SMV/IFN/RBV	–	–	–	–	–	97.8	–
	Pre DCV/ASV	99.3	–	–	–	–	98.7	–
	Post DCV/ASV	99.7	–	–	78.3	99.5	99.3	–
	18 M after DCV/ASV	78.9	–	–	1.4	74.0	78.8	–
LT #4	Pre SMV/IFN/RBV	–	–	–	–	–	–	99.9
	Post SMV/IFN/RBV	83.4	33.3	–	–	–	–	35.3
	Pre DCV/ASV	35.1	28.8	–	–	–	–	40.5
	Post DCV/ASV	89.6	–	–	6.7	92.4	–	6.4
	18 M after DCV/ASV	–	–	–	96.8	45.9	–	56.2

*LT* liver transplant, *SMV/IFN/RBV* simeprevir plus interferon plus rivabirin, *DCV/ASV* daclatasvir/asunaprevir, – not detectable.

The results of the phylogenetic analysis of the four LT patients are provided in Fig. 2. In LT patient #1 and #2, the phylogenetic trees of the HCV clones before and after DAA treatment were found to be similar to those of four non-LT patients reported in our previous paper^14^. In these two patients, a part of the HCV clones before DAA treatment acquired RASs, and the HCV clones with RASs were selected and proliferated during the DAA administration (Fig. 2A,B). Surprisingly, phylogenetic tree analysis in LT patient #3 and #4, who acquired NS5A P32 deletion, demonstrated patterns that were different from those of the other two LT patients and four non-LT patients (Fig. 2C,D). The HCV clones were widely distributed, and HCV clones with NS5A P32 deletion were found to emerge from multiple clones. The wide distribution of the HCV clones in LT patient #3 and #4 continued to spread, even during the natural course, for 18 months after the termination of DAA treatment.

### Mutational spectrum in patients during DAA treatment, interferon-based treatment, and natural courses after DAA treatment

**Figure 3:**
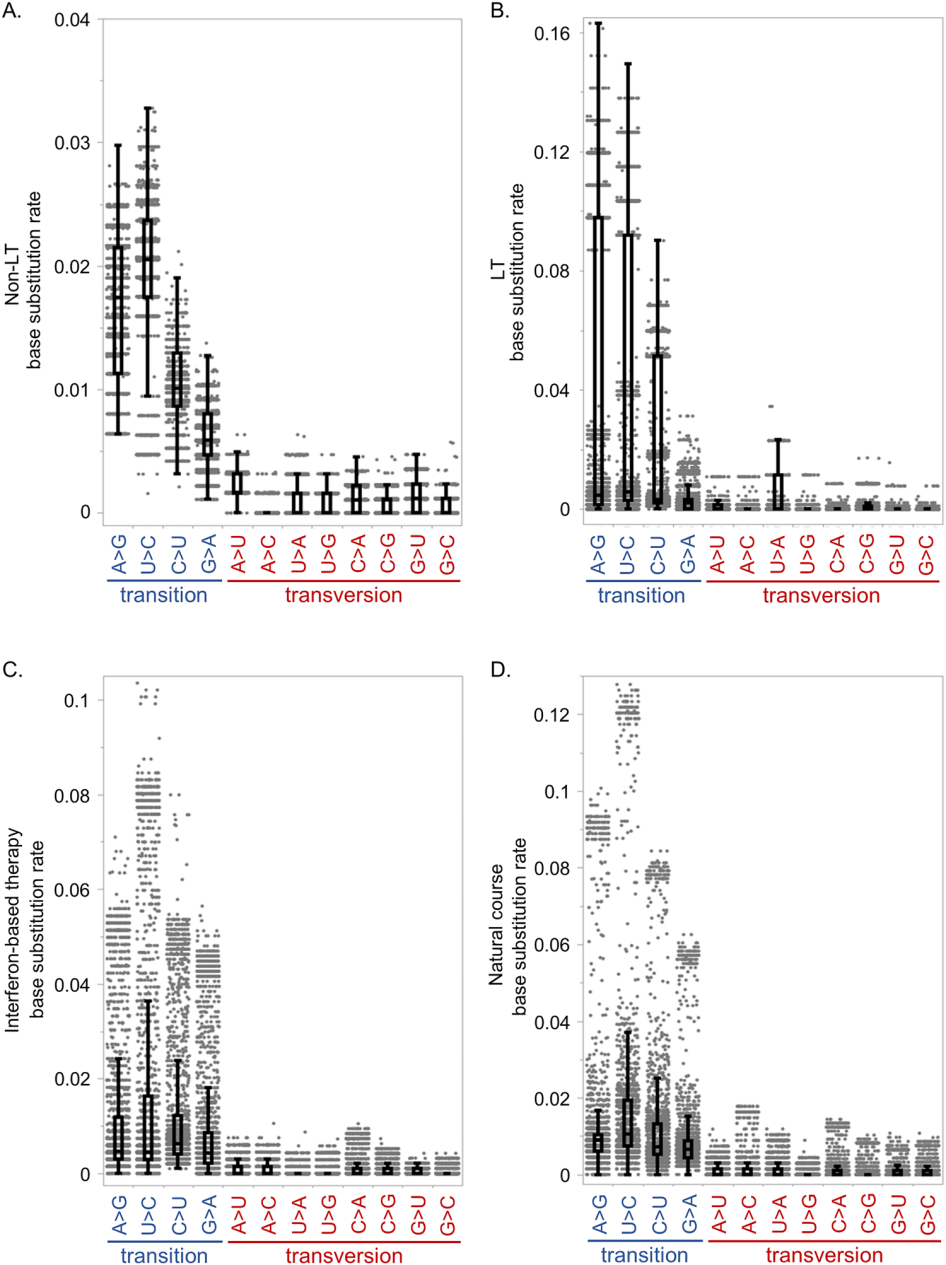
Mutational spectra of patients during DAA treatment, interferon-based treatment, and natural courses after DAA treatment. Base substitution rates during DAA treatment in four non-liver transplant (Non-LT) patients (**A**) and four post-liver transplant (LT) patients (**B**); and during simeprevir, interferon and ribavirin therapy (**C**), and 18 months’ natural course after DAA treatment (**D**) in four post-liver transplant patients.

A mutational spectrum was observed during DAA treatment in four non-LT patients analyzed using SMRT sequencing. In the 12 possible base substitutions, the frequency of transitions was significantly more frequent than transversions. Moreover, the A>G and U>C transitions were more frequent than the G>A and C>U transitions (Fig. 3A). Interestingly, this mutational bias is identical to that found in the HCV replicon system (Fig. 1), indicating that the mutational spectra of patients is similar to those in replicon cell lines even during DAA treatment. Moreover, the mutational patterns observed in non-LT patients were similar to those in LT patients (Fig. 3B), indicating that immunosuppressive agents used in transplant recipients were not affected in terms of establishing HCV mutation bias, although the mutational effect by the previous treatment regimen might not completely be excluded.

**Figure 4:**
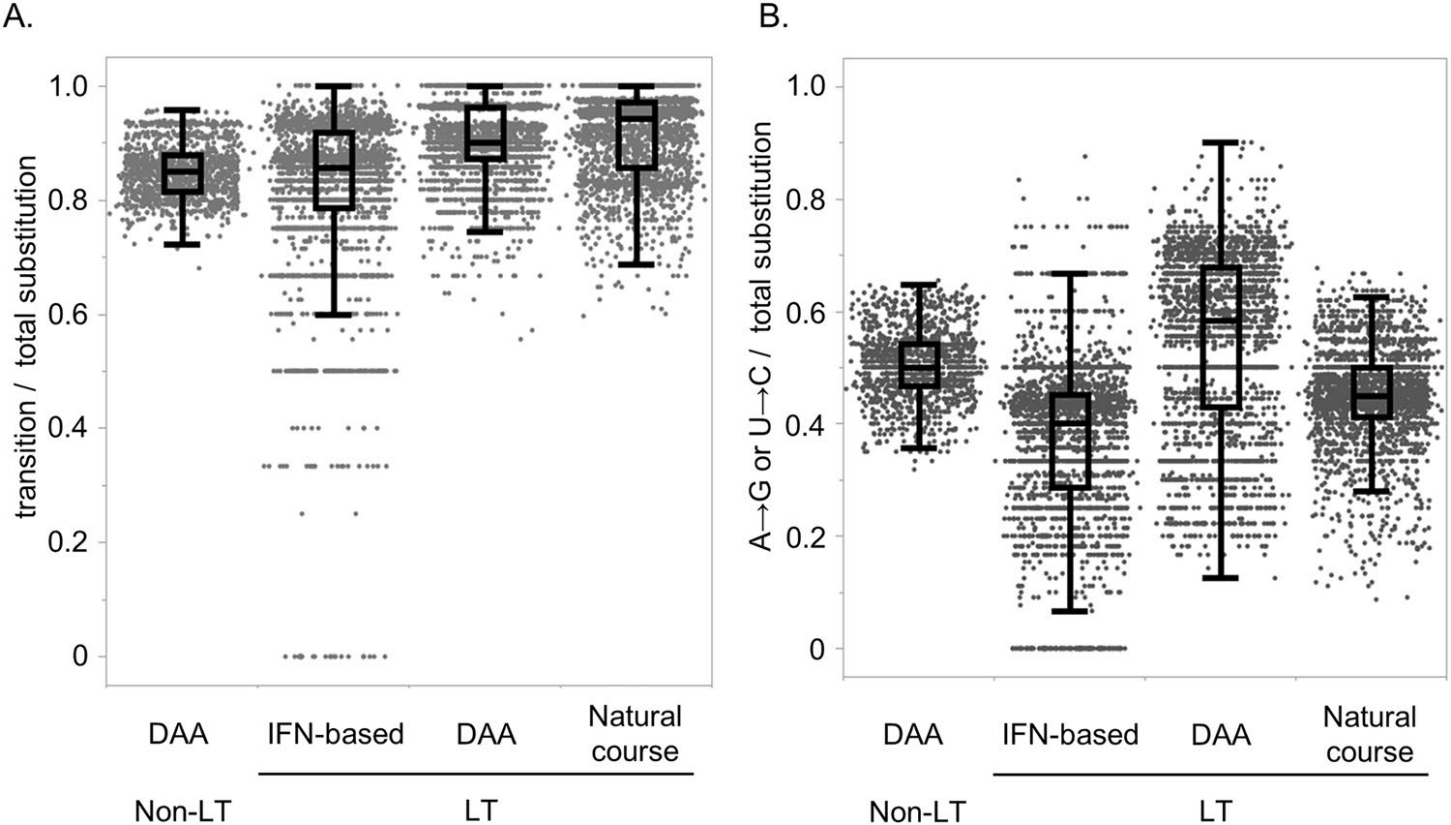
Mutational spectra of HCV genome in patients with chronic HCV infection. Rates of transition substitutions to total substitutions (**A**), and A>G and U>C transitions to total substitutions (**B**) during direct-acting antiviral therapy (DAA) in four non-liver transplant (Non-LT) patients, and during DAA, interferon (IFN)-based therapy, and natural course after DAA treatment in four post-liver transplant (LT) patients.

In addition, during interferon-based therapy with SMV/PegIFN/RBV and natural courses after DAA treatment, transition substitutions were significantly more frequent than transversions (Fig. 3C,D). Transitions yielded over 80% of the total substitutions during DAA therapy in both non-LT and transplant patients, as well as during interferon-based therapy and natural course after DAA treatment (Fig. 4A). During natural courses after DAA treatment, the A>G and U>C transitions were significantly more frequent than the C>U and G>A transitions (Figs. 3D, 4B). NS5A P32 deletion would not affect the mutational patterns, because no apparent differences of the substitution patterns between two patients without NS5A P32 deletion (LT patient #1 and #2) and two patients with P32 deletion (LT patient #3 and #4) was shown (Supplementary Figs. 2, 3). However, during interferon-based therapy, the mutational pattern varied from that during DAA treatment or natural courses after DAA treatment (Fig. 3C). The C>U transition was the most frequent among the substitutions, suggesting that the administration of ribavirin, which is a synthetic guanosine nucleotide, affected the mutational pattern.

### Mutational pattern for emergence of RASs during DAA treatment

**Figure 5:**
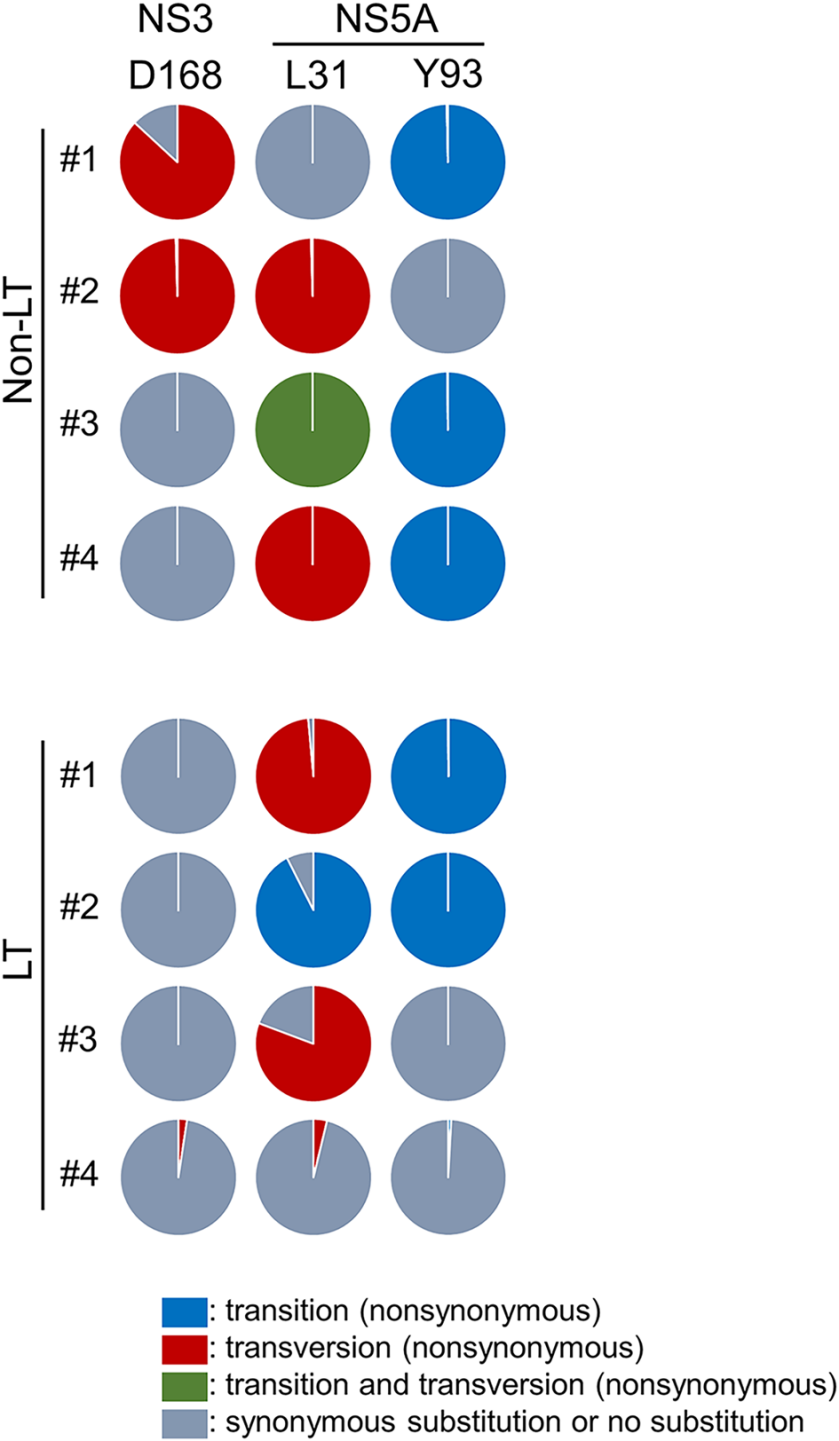
Mutational patterns for emergence of resistance-associated substitutions from Pre daclatasvir and asunaprevir (DCV/ASV) to Post DCV/ASV. Transition and transversion substitutions at NS3 D168 and NS5A L31 and Y93 in non-LT patients #1–4 and in LT patients #1–4 are shown in each pie chart. Mutations from Pre DCV/ASV to Post DCV/ASV were identified in each patient based on the phylogenetic analysis. The nucleotide sequences of an HCV clone at Pre DCV/ASV with the nearest clones of genetic distance from the HCV clones at Post DCV/ASV was determined as the reference sequence in each case, and all the sequences of HCV clones at Post DCV/ASV were compared with each reference sequence, where the base substitutions were identified as mutations acquired from Pre DCV/ASV to Post DCV/ASV. The frequencies of transition and transversion substitutions in each locus are shown. LT, liver transplant.

Next, we analyzed the mutational patterns at each nucleotide positions of the HCV genome in the eight patients after DAA treatment by comparing them to the patients before treatment. Although the majority of mutations were transitions, several mutations occurred by transversion substitution. We hypothesized that nonsynonymous mutations occurred by much less frequent transversion substitutions during DAA therapy would be meaningful mutations that were selected and proliferated under DAA treatment pressure. Therefore, we investigated the nonsynonymous mutations that occurred by transversion substitution, and found that they accounted for over 10% of the total clones after DAA administration, observed in more than one of the eight patients. Only two nucleotide positions were found to fulfil these criteria, which resulted in amino acid substitutions at NS3 D168 and NS5A L31, which are known as RASs for the NS3/4A and NS5A inhibitors, respectively. NS3 D168V substitutions occurred as a result of GAC>GUC transversion substitutions in non-LT patient #1 and #2 (Fig. 5). In LT patient #4, most of the HCV clones with NS3 D168 substitutions were expanded from pre-existing HCV clones with NS3 D168V and D168E. However, minor clones with D168V and D168E arose due to the GAA>GUU transversion (1.54%) and GAU>GAA transversion (0.85%), respectively. NS5A L31 substitutions occurred as a result of the following nucleotide substitutions: UUG>GUG (transversion) in non-LT patient #2 (L31V); CUG>AUA (transition and transversion) in non-LT patient #3 (L31I); UUG>AUG (transversion) in non-LT patient #4 (L31M); CUG>GUG (transversion) in LT patient #1 (L31V); AUG>GUG (transition) in LT patient #2 (M31V); UUA>UUU (transversion) in LT patient #3 (L31F) (Fig. 5). In LT patient #4, minor clones with L31M arose as a result of the CUG>AUG transversion (2.74%) and UUG>AUG transversion (1.03%). In addition, NS5A Q54H substitution occurred due to CAG>CAU transversion substitutions in non-LT patient #3 (data not shown). HCV clones with NS3 D168V, NS5A L31V/I/M, and Q54H mutations yielded over 78% (78.3 to 100%) of all HCV clones after DAA treatment, except for the HCV clones with NS3 D168V/E and NS5A L31M in LT patient #4 (Table 3), suggesting that the HCV clones with these RASs were selected under DAA treatment pressure. All of the Y93H substitutions found in five of the eight patients occurred as a result of UAC>CAC transition mutations. Moreover, over 99% (99.54 to 100%) of all HCV clones showed Y93H RAS after DAA treatment in these five patients (Fig. 5). NS3 Q80R and NS5A R30Q in non-LT patient #4 also occurred as a result of transition mutations (data not shown).

These results suggest that RASs were produced by both transition and transversion, which were selected and proliferated under the selective pressure of DAA treatment. Since the transition substitutions occurred much more frequently in HCV patients, the nonsynonymous transversion substitutions selected after DAA treatment are likely to be meaningful substitutions for drug resistance and/or viral proliferation.

## Discussion

In this study, we investigated the HCV mutational spectrum in patients with chronic HCV infection, both in non-transplant and post-transplant settings before and after anti-HCV treatment using SMRT sequencing. Long-read and high accuracy sequencing techniques revealed that, due to HCV mutational bias, transition mutations were more frequent than transversion, and the A>G and U>C substitutions were predominant in the transition mutations in the HCV genome in patients in both non-transplant and post-transplant settings both during DAA treatment and during natural courses after DAA treatment. Moreover, nonsynonymous substitutions were found to occur by transversion mutations that survived under selective pressure, including RASs of NS3 D168E/V and NS5A L31V/I/M/F during DAA treatment.

In the current study, we revealed the mutational spectrum in the HCV genome of patients during DAA treatment, interferon-based treatment, and natural course after DAA treatment. Surprisingly, the mutation patterns in patients were almost identical to those observed in HCV replicon cell lines. Transition mutations, especially A>G and U>C substitutions, predominantly occurred in both immunosuppressive and immunocompetent patients, as well as both under DAA treatment pressure and during natural courses after DAA treatment in patients with chronic HCV infection. Interestingly, after treatment with SMV/PegIFN/RBV, the mutational patterns in transition substitutions were different from those under other situations (i.e. C>U transition was the most frequent substitution among transitions). Previous reports have demonstrated that ribavirin induces C>U and G>A substitutions in HCV genome^24,26,27^. Therefore, the alterative mutation pattern during interferon-based therapy may be caused by the administration of ribavirin.

The higher frequency of transition mutations compared to transversion mutations may be explained by viral polymerase fidelity. The same mutation patterns among in vitro biochemical analysis in HCV replicon cells and HCV-infected patients^20,22,24^ suggests that the RdRp fidelity of HCV NS5B is a major factor in establishing the mutational spectrums of HCV RNA. Moreover, the higher frequency of the A>G and U>C than G>A and C>U transition substitutions are also thought to arise from the fidelity of HCV NS5B. Interestingly, in poliovirus, an RNA virus of a member of Enterovirus C virus in the Picornaviridae family, transition substitutions are more frequent than transversion substitutions, as in HCV. However, the transitions G>A and C>U are more frequent than A>G and U>C in vivo, which is different from the mutational patterns observed in HCV^28^. In human immunodeficiency virus 1 (HIV1), G>A substitutions are the most frequent type of substitution in the RNA, due to the apolipoprotein B mRNA-editing enzyme catalytic polypeptide-like 3 (A3)-mediated cytidine deamination and the fidelity of the viral reverse transcriptase^29,30^. These differences in mutational preference among viruses indicate that the fidelity of RNA polymerase is dependent on the characteristics of each virus.

Although a significant bias toward transition substitution mutations was found in HCV-infected patients, some RASs emerged as a result of transversion substitutions of the HCV RNA, suggesting that HCV with rare transversion mutations at specific nucleotide positions were selected and proliferated under DAA treatment pressure. Conversely, the selected nonsynonymous mutations occurred by transversions in patients are likely to be meaningful mutations for viral survival. In fact, only NS3 D168E/V and NS5A L31V/I/M/F, which are major RASs against HCV protease inhibitors and NS5A inhibitors, respectively, were identified as nonsynonymous substitutions by transversion mutations during DAA treatment. There are of course some exceptions and the mutational pattern depends on the original HCV sequence, as shown in LT patient #2, in whom NS5A M31V occurred by transition substitution. However, nonsynonymous substitutions by transversion mutations, NS3 D168E and NS5A L31M/I/F in natural course after DAA treatment, and NS3 D168V/A/E during interferon-based therapy, found in the current study, may be essential for viral survival.

According to the result of the phylogenetic analysis (right panels in Fig. 2), we found that the HCV clones were dramatically changed during the natural course after DAA treatment in all four LT patients. A part of the HCV clones presented just after DAA treatment that acquired several other substitutions in the HCV genome were selected and proliferated as different clusters in phylogenetic trees. The results of SMRT sequencing demonstrated that HCV mutations occurred constantly, and some clones with growth advantages were found to be selected and proliferated under different patient circumstances, such as interferon-based therapy, all oral DAA treatment, and natural course after DAA treatment.

In the present study, two patients acquired the NS5A P32 deletion after DAA treatment. This deletion is a potent RAS associated with DAA treatment failure in patients^6,31–34^. The two patients who had HCV with NS5A P32 deletion demonstrated much a higher diversity of HCV than the other six patients, according to phylogenetic tree analysis. One patient (LT patient #4) showed a great diversity of HCV even before DAA treatment, when HCV with NS5A P32 deletion was not present, indicating that the genomic instability of HCV of this patient was caused by neither DAA treatment nor the deletion of NS5A P32 itself. Thus, we speculated that the HCV in this patient acquired genomic instability for an unknown reason, and that the genomic instability resulted in many mutations in the HCV genome, including the NS5A P32 deletion. However, since only two cases with HCV NS5A P32 deletion were analyzed, further study will be needed to clarify the mechanism of genomic instability in patients with the NS5A P32 deletion.

In conclusion, the RdRp fidelity of HCV NS5B produces mutational bias in the HCV genome, predominantly characterized by transition mutations, especially A>G and U>C substitutions, in patients with chronic HCV infection. In contrast, RASs at NS3 D168 and NS5A L31 were found to be acquired mainly via transversion substitutions, indicating that the HCV clones with RASs arise via both transition and transversion mutations, and are selected for and proliferate under DAA treatment pressure.

## Methods

### HCV replicon cell lines

HCV replicon cell lines derived from HuH-7 cells were established as described previously^25,35^, and cultured for 10 years after establishment in Dulbecco’s modified Eagle’s medium supplemented with 10% fetal bovine serum and G418 (0.3 mg/ml)^23^. These replicon cell lines were passaged every 7 days. RNA samples extracted from the two HCV replicon cell lines at establishment (year 0) and after 10 years’ culture (year 10) were analyzed.

### Patients

Four patients with HCV infection after liver transplantation, who were assigned to receive all oral DAA therapy at Kyoto University and resulted in non-SVR from September 2015 to December 2018, were enrolled in this study. All four patients have experienced treatment failure with interferon-based regimen and then experienced treatment failure with daclatasvir (60 mg/day) and asunaprevir (200 mg/day) (DCV/ASV) for 24 weeks. Serum samples were collected from the four patients before interferon-based therapy, at treatment failure of interferon-based therapy, before all oral DAA treatment, at treatment failure of DAA treatment, and 18 months after DAA failure. The interferon-based therapies in the four patients were triple therapy with simeprevir (100 mg/day), peginterferon, and ribavirin (SMV/PegIFN/RBV) for the first 12 weeks, followed by dual therapy with peginterferon and ribavirin for another 12 weeks. Serum samples before DAA treatment and at treatment failure were also collected from the four non-LT patients who had treatment failure with 24 week-treatment of DCV/ASV. The serum HCV RNA load was evaluated using a real-time PCR-based quantification method for HCV (COBAS AmpliPrep/COBAS TaqMan HCV Test, Roche Molecular Systems, Pleasanton, CA, USA).

All protocols were approved by the Ethics Committee of Kyoto University. Serum samples and clinical information were obtained with written informed consent based on the Study Protocol R0217. The protocol of this study complied with all provisions of the Declaration of Helsinki.

### Single molecular real-time (SMRT) sequencing

SMRT sequencing was conducted using a PacBio RSII and Sequel sequencers, according to the manufacturer’s protocol (Pacific BioScience, Menlo Park, CA, USA)^9,10,36^. Briefly, total RNA was extracted from the serum using a QIAquick Viral Mini kit (Qiagen, Valencia, CA, USA) and from cell lines using a Qiagen RNeazy mini kit and Qiagen shredder (Qiagen, Valencia, CA, USA). HCV sequences spanning the NS3 and NS5A regions (3120 base) of the HCV genome for the serum samples and spanning NS3 to NS5B (5972 base) for the HCV replicon cell lines were amplified using a PrimeScript One Step RT-PCR kit (Takara Bio, Shiga, Japan) and PrimeSTAR HS kit (Takara Bio, Shiga, Japan) using the HCV-specific primers shown in Supplementary Table 2. The purified DNA product (5 µg) was used to construct the PacBio DNA library, according to the PacBio standard template prep protocol. The samples were sequenced on the PacBio RSII or Sequel platform on a single SMRT cell per sample. P6C4 polymerase was used for the sequencing reaction and 6-h movie windows were used for signal detection. After raw sequence data were generated, base-calling and CCS read generation were performed using version 2.3.0.1 of PacBio’s instrument control and SMRT analysis software with default parameters^12,37^. To ensure a high accuracy of the sequence reads, we selected CCS reads with at least 5 passes around the closed loop SMRTbells (5-pass CCS reads). The output sequences are provided in FASTQ format.

To evaluate the RAS frequencies, we analyzed the sequence data 5- or more pass CCS reads using LastZ (version 1.0.4) at Galaxy (https://usegalaxy.org/)^38^. The sequence data were uploaded to the Galaxy web platform and aligned with the reference sequences for the entire HCV genome (GenBank: D90106.1) using the Galaxy public server^39^.

To generate phylogenetic trees and evaluate the mutational spectrum in the HCV genome, long CCS reads (> 3000 bp, in one case > 2000 bp) were selected using R (version 2.2.1). The selected 10- or more pass CCS reads in the non-LT patients in FASTA format were aligned in Clustal W. In the post-transplantation patients, we selected about 500 viral isolate sequences in descending order of CCS pass number in 5- or more pass CCS reads (Table 1). Phylogenetic analysis and mutational landscape analysis were conducted using these sequence reads. The evolutionary history was inferred using the Maximum Likelihood method. Evolutionary analyses were conducted in MEGA7^40^.

### Control experiment to confirm the accuracy of SMRT sequencing

As a control experiment, we first sequenced the NS3/4 and NS5A regions amplified from the HCV-containing plasmid as a template. The SMRT sequencing platform provided a total of 127,200 continuous raw reads covering 3946 bp of the NS3/4 and 5A genome regions derived from the plasmids. Among the total CCS reads generated with the custom PacBio RSII program, we analyzed the error rate of each pass CCS read (Supplementary Table 3). As a result, the mean mismatch error rate of 5- or more pass CCS reads was 0.000252 (SD = 0.000192) per base pair. This indicated an extremely high accuracy of sequence reads achieved by the current SMRT sequencing platform by selecting 5- or more pass CCS reads. Therefore, we used the sequence data with 5- or more pass CCS reads in the subsequent analyses.

### Determination of nucleotide substitutions at single viral clone resolution

In the cell line experiments, the original HCV replicon sequence^25^ was used as the reference sequence for the detection of mutations. Each 5 or more pass CCS read of HCV sequence extracted from HCV replicon cell line at Year 0 was compared with this reference sequence, where the base substitutions were identified as mutations.

In the comparative analysis of HCV clones at Year 0 and Year 10, the genetic distances between every two clones at different time points were calculated using Tamura-Nei model^40^. Then, the HCV clone at Year 0 which is genetically nearest from each clone at Year 10 was selected as the reference clone, and the nucleotide differences between each clone at Year 10 and the reference clone at Year 0 were identified to be mutations occurring during the 10-year culture.

Similarly, for the analyses of clinical samples, mutations were identified between two different time points in patients based on the phylogenetic analysis. A HCV clone at first time point with the nearest clones of genetic distance from the HCV clones at a second time point was selected as the reference, and the paired sequences of each HCV clone at a second time point and the reference clone were compared, where the base substitutions were identified as mutations. Variants found in less than 1% of the total HCV clones at a time point were excluded from the phylogenetic analysis.

### Analysis of mutational spectrum of HCV genome using data from the SMRT sequencing

All base substitutions detected were classified into transition substitutions (or transitions) and transversion substitutions (transversions). Transitions include purine (A/G) to purine, or pyrimidine (C/U) to pyrimidine substitutions, which consist of A>G, G>A, U>C, and C>U substitutions, while transversions include the other substitution patterns. By using the SMRT sequencing data, we analyzed the mutational spectrum during 10 years in the HCV replicon system and in the HCV clones obtained from sera samples of patients with chronic HCV infection. The rates of transition substitutions to the total substitution at each nucleotide position were demonstrated as transition/total substitutions.

The base substitution rate of C>U in a single viral clone was defined as follows

$$\frac{The\;number\;of\;C > U\;site\;in\;the\;clone\;analyzed}{A\;total\;number\;of\;C\;contained\;in\;the\;reference\;sequence.}$$

The base substitution rates of the other nucleotide substitutions were similarly defined. The base substitution rates of a total of 12 substitution patterns, such as C>U, C>A, C>G, U>C, U>A and U>G, were calculated per single viral clone.

### Evolutionary analysis by Maximum Likelihood method

The evolutionary history was inferred by using the Maximum Likelihood method and Tamura-Nei model. The tree with the highest log likelihood is constructed. Initial tree for the heuristic search were obtained automatically by applying Neighbor-Join and BioNJ algorithms to a matrix of pairwise distances estimated using the Tamura-Nei model, and then selecting the topology with superior log likelihood value^40^. The trees were drawn to scale, with branch lengths measured in the number of substitutions per site. All five- or more pass CCS reads analyzed at each time point were used for the phylogenetic analysis, and each phylogenetic tree was constructed using sequence information of CCS reads at two different time points. Evolutionary analyses were conducted in MEGA 7 software^40^.

### Statistical analysis

To compare the rate of base substitution patterns in the HCV clones during SMV/PegIFN/RBV treatment, DCV/ASV treatment, and 18 months natural course after DAA treatment, continuous variables were analyzed using the Wilcoxon signed-rank test. Data were analyzed using JMP pro (version 14.0.0). Two-tailed probability values of *p* < 0.05 were considered significant.

## Supplementary Information

Supplementary Information.

## Data Availability

The sequence reads are available at the DNA Data Bank of Japan Sequence Read Archive under accession number DRA009132.

## Abbreviations

CCS: Circular consensus sequencing
DAA: Direct acting antiviral
DCV/ASV: Daclatasvir and asunaprevir
LT: Liver transplant
RAS: Resistance-associated substitution
RdRp: RNA-dependent RNA polymerase
SMRT: Single molecule real-time
SMV/PegIFN/RBV: Simeprevir, peginterferon, and ribavirin
SVR: Sustained virologic response
