# Supplementary material for: Mutational spectrum of hepatitis C virus in patients with chronic hepatitis C determined by single molecule real-time sequencing
Source: Sci Rep. 2022 Apr 30;12:7083. doi: 10.1038/s41598-022-11151-6 (PMC9056513; doi:10.1038/s41598-022-11151-6)
Supplement: Supplementary file 1 — Supplementary Information. [file 41598_2022_11151_MOESM1_ESM.docx]

**Supplementary Materials**

**Mutational spectrum of hepatitis C virus in patients with chronic hepatitis C determined by single molecule real-time sequencing**

Fumiyasu Nakamura^1,¶^, Haruhiko Takeda^1,¶^, Yoshihide Ueda^1,2,＊^, Atsushi Takai^1^, Ken Takahashi^1^, Yuji Eso^1^, Soichi Arasawa^1^, Eriko Iguchi^1^, Takahiro Shimizu^1^, Masako Mishima^1^, Ken Kumagai^1^, Taiki Yamashita^3^, Shinji Uemoto^4^, Nobuyuki Kato^5^, Hiroyuki Marusawa^1^, Akihiro Sekine^3^, Hiroshi Seno^1^

^1.^ Department of Gastroenterology and Hepatology, Graduate School of Medicine, Kyoto University, Kyoto, Japan

^2.^ Division of Gastroenterology, Department of Internal Medicine, Kobe University Graduate School of Medicine, Kobe, Japan.

^3.^ Center of Preventive Medical Sciences, Chiba University, Chiba, Japan

^4.^ Department of Surgery, Graduate School of Medicine, Kyoto University, Kyoto, Japan

^5.^ Department of Tumor Virology, Okayama University Graduate School of Medicine, Dentistry, and Pharmaceutical Sciences, Okayama, Japan

¶ These authors contributed equally to this work.

Contents

Supplementary Figures 1-3

Supplementary Tables 1-3

**Supplementary Figure 1**

**
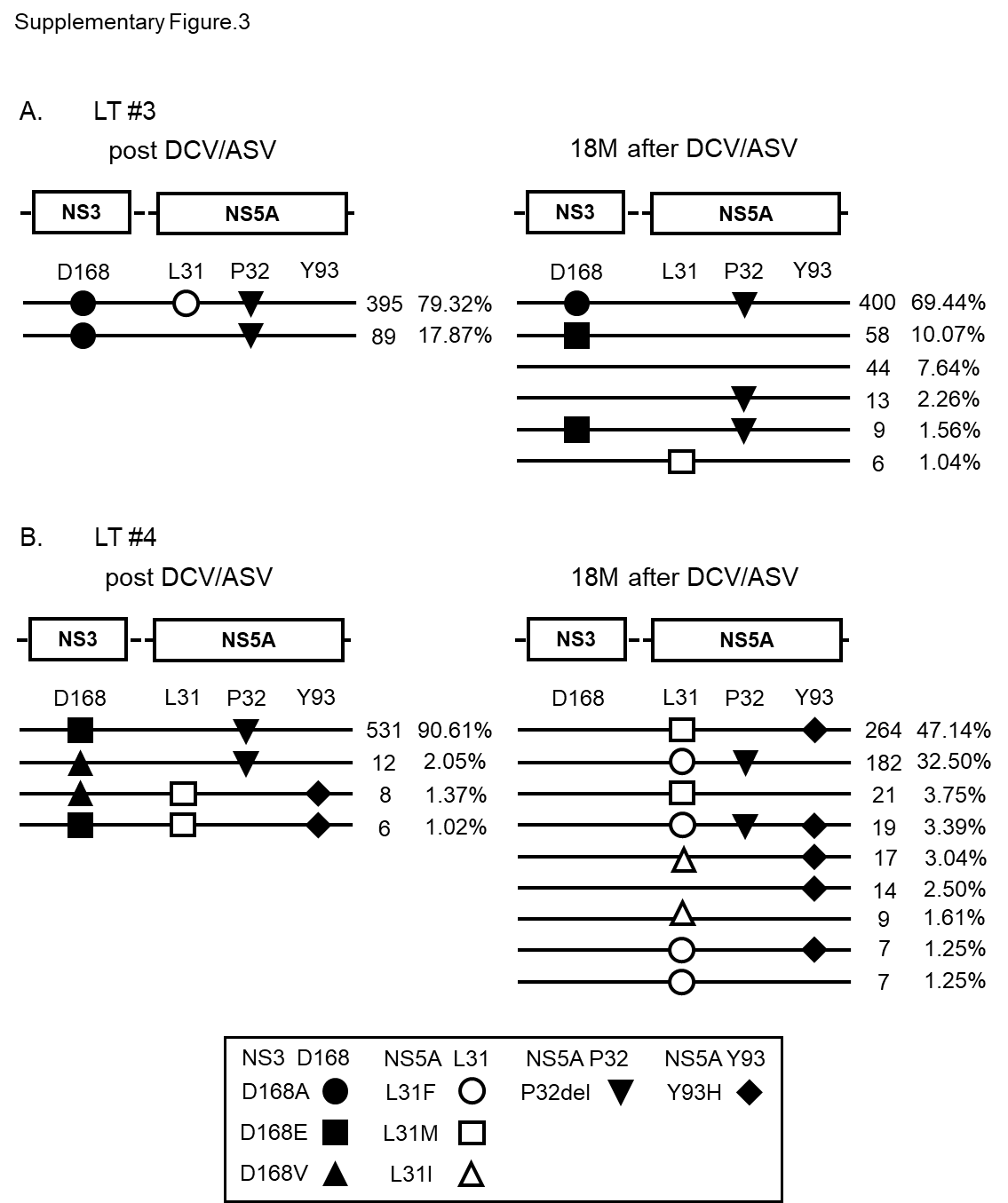
**

**Supplementary Figure 1. Linkage analysis of multiple RASs on the identical HCV clones.** Linkage of multiple resistance-associated substitutions (RASs), including the substitutions at NS3 D168, NS5A L31, P32 and Y93 are shown. A and B describe LT #3 and LT #4. In each panel, NS3 and NS5A regions of HCV genome are schematically described. Horizontal lines demonstrate CCS reads of HCV clones with any RASs, that is, the haplotypes of HCV clones existing at each timepoint. Two parameters on the right of each read show the CCS read number and the frequency of each haplotype. Frequency of each haplotype was calculated as the number of CCS reads with each RAS-based haplotype among all viral clones determined by SMRT sequencing at each timepoint. Only the major haplotypes with frequencies of more than 1 % are described here.

**Supplementary Figure 2**


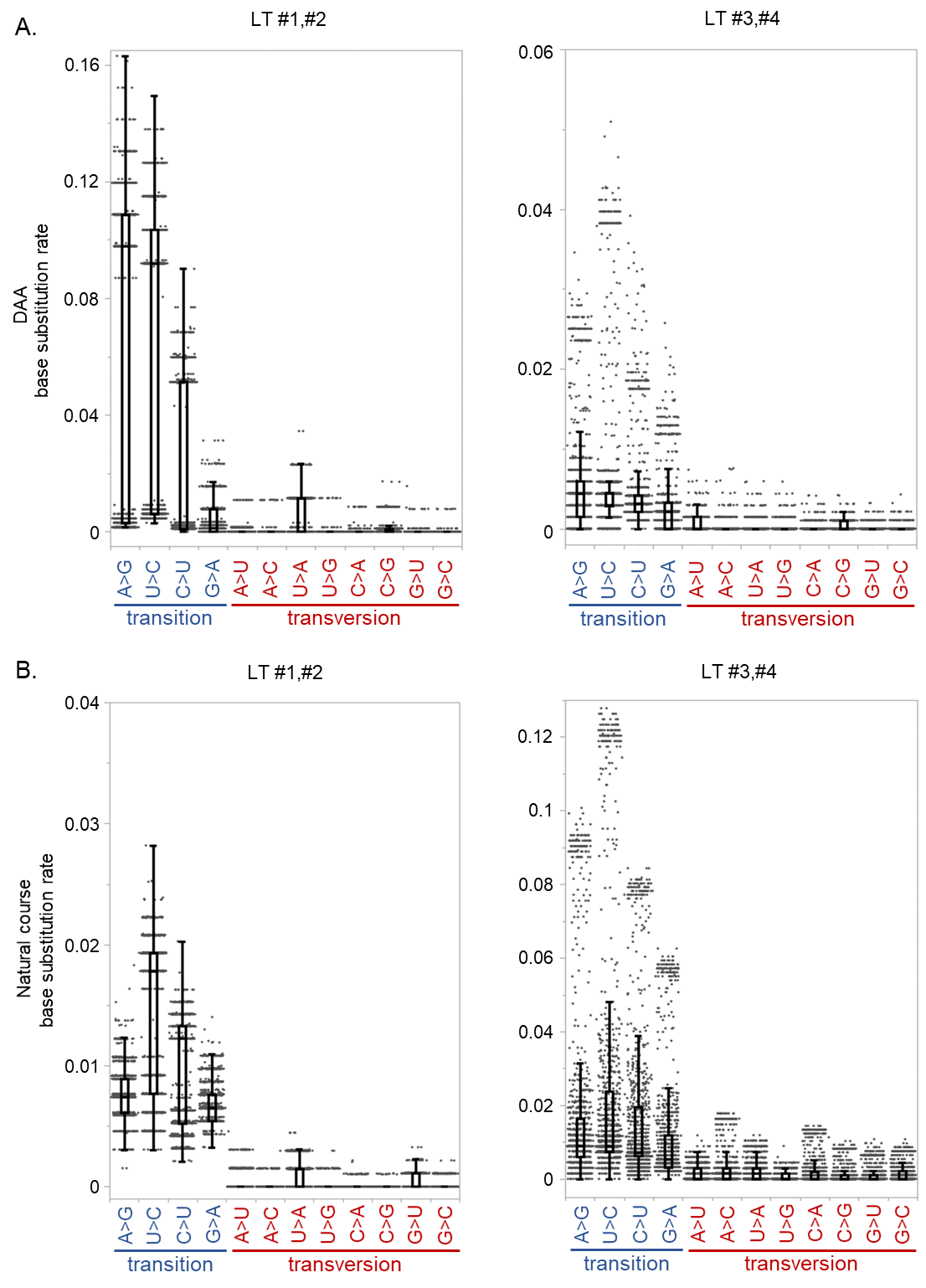


**Supplementary Figure 2 Mutational spectra of patients during DAA treatment and natural courses after DAA treatment according to the presence of NS5A P32 deletion.**

**A.** Dot and box plots demonstrate the base substitution rates during DCV/ASV treatment in four post-liver transplant (LT) patients. Left panel; LT#1 an #2 without NS5A P32 deletion. Right panel; LT#3 and #4 with NS5A P32 deletion. **B.** Dot and box plots demonstrate the base substitution rates of 4 LT patients 18 months’ natural course after DCV/ASV treatment.

**Supplementary Figure 3**

**
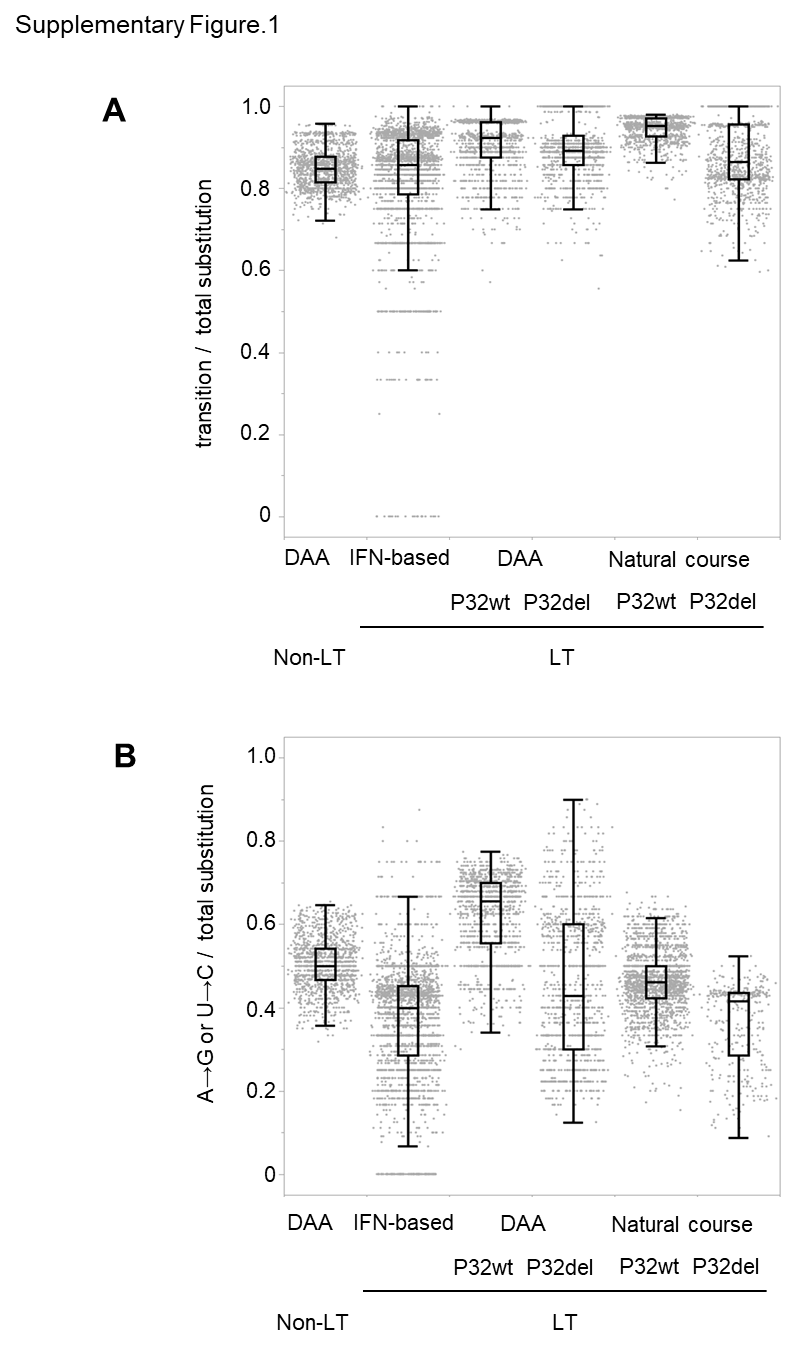
**

**Supplementary Figure 3 Mutational spectra of HCV genome in patients with chronic HCV infection according to the presence of NS5A P32deletion.**

Rates of transition substitutions to total substitutions (A), and A>G and U>C transitions to total substitutions (B) during direct-acting antiviral therapy (DAA) in four non-liver transplant (Non-LT) patients, and during DAA, interferon (IFN)-based therapy, and natural course after DAA treatment in four post-liver transplant (LT) patients. LT patients were divided into two groups, with or without NS5A P32 deletion (labeled with P32del and P32wt).

**Supplementary Table 1**

**Amino acid substitutions in HCV replicons during 10years’ culture**

**a) HCV replicon line1**

|  | | 0year | 10years |  |  | | 0year | 10years |  |  | | 0year | 10years |  |
| --- | --- | --- | --- | --- | --- | --- | --- | --- | --- | --- | --- | --- | --- | --- |
| NS3 | 9 | Q | R | 99.72% | NS5A | 37 | L | F | 97.65% | NS5A | 380 | F | V | 98.48% |
|  | 41 | Q | R | 95.57% |  | 44 | K | R | 10.80% |  | 382 | S | N | 96.81% |
|  | 61 | S | P | 98.75% |  | 78 | K | R | 22.99% |  | 385 | S | P | 99.86% |
|  | 71 | I | V | 99.58% |  | 132 | G | A | 99.72% |  | 392 | T | I | 15.79% |
|  | 86 | Q | R | 97.51% |  | 139 | K | R | 99.17% |  | 393 | A | V | 98.06% |
|  | 122 | N | S | 99.86% |  | 215 | K | R | 42.38% |  | 398 | D | A | 21.33% |
|  | 179 | M | T | 98.48% |  | 248 | D | H | 99.45% |  | 402 | D | G | 99.31% |
|  | 196 | T | A | 99.86% |  | 257 | A | D | 98.75% |  | 403 | D | G | 98.89% |
|  | 288 | V | I | 88.64% |  | 265 | M | V | 98.89% |  | 408 | S | P | 99.45% |
|  | 297 | S | P | 99.58% |  | 266 | G | R | 99.45% |  | 409 | D | G | 97.78% |
|  | 305 | T | S | 98.89% |  | 273 | E | G | 96.12% |  | 416 | M | V | 98.75% |
|  | 365 | V | L | 88.92% |  | 295 | E | K | 87.12% |  | 419 | L | P | 84.35% |
|  | 379 | T | I | 93.35% |  | 297 | S | P | 99.17% |  | 420 | E | G | 94.74% |
|  | 387 | N | H | 98.75% |  | 301 | E | G | 97.92% |  | 434 | S | A | 98.61% |
|  | 390 | A | V | 99.72% |  | 305 | K | E | 99.31% |  | 437 | S | T | 99.17% |
|  | 430 | T | I | 96.26% |  | 306 | T | S | 95.43% |  | 446 | C | R | 99.17% |
|  | 449 | T | A | 99.58% |  | 308 | K | E | 96.68% | NS5B | 81 | K | R | 98.34% |
|  | 511 | V | A | 98.34% |  | 315 | I | V | 18.70% |  | 117 | N | D | 68.98% |
|  | 555 | D | E | 99.45% |  | 326 | L | S | 99.86% |  |  | N | G | 24.10% |
|  | 580 | Q | R | 99.45% |  | 328 | S | P | 98.06% |  | 247 | S | P | 86.01% |
|  | 583 | K | E | 95.57% |  | 348 | K | T | 99.45% |  | 315 | A | V | 88.64% |
|  | 593 | H | R | 13.02% |  | 357 | R | G | 95.84% |  | 335 | S | R | 99.72% |
| NS4A | 29 | I | V | 99.31% |  | 358 | K | E | 93.63% |  | 441 | K | I | 24.10% |
|  | 46 | Q | R | 99.58% |  | 362 | I | V | 99.45% |  | 520 | T | M | 96.54% |
| NS4B | 46 | L | F | 10.39% |  | 366 | S | P | 99.58% |  | 540 | P | A | 56.51% |
|  | 50 | W | L | 97.23% |  | 367 | T | A | 99.03% |  | 566 | R | H | 53.19% |
|  | 161 | E | V | 98.06% |  | 377 | T | A | 99.58% |  | 572 | F | L | 99.45% |
|  | 162 | M | V | 99.58% |  | 378 | K | E | 97.92% |  | 585 | I | T | 95.43% |

**b) HCV replicon line2**

|  | | 0year | 10years |  |  | | 0year | 10years |  |  | | 0year | 10years |  |
| --- | --- | --- | --- | --- | --- | --- | --- | --- | --- | --- | --- | --- | --- | --- |
| NS3 | 29 | V | I | 99.39% | NS5A | 68 | K | R | 31.13% | NS5A | 393 | V | A | 26.99% |
|  | 30 | E | A | 97.85% |  | 122 | T | M | 23.62% |  | 400 | A | T | 98.62% |
|  | 80 | Q | R | 99.23% |  | 157 | Q | R | 57.36% |  | 401 | S | A | 98.93% |
|  | 174 | S | C | 62.12% |  | 198 | V | A | 35.58% |  | 402 | D | V | 98.31% |
|  | 259 | I | L | 99.69% |  | 207 | S | P | 99.39% |  | 405 | D | G | 52.76% |
|  | 272 | E | K | 50.46% |  | 239 | L | V | 32.36% |  | 406 | K | E | 94.17% |
|  | 357 | E | Q | 99.69% |  | 240 | K | Q | 99.23% |  | 409 | D | G | 37.42% |
|  | 394 | V | G | 34.82% |  | 246 | H | R | 10.74% |  | 410 | V | D | 99.69% |
|  | 436 | V | A | 100.0% |  | 248 | D | G | 22.24% |  | 419 | L | P | 72.70% |
|  | 513 | V | L | 50.61% |  | 282 | E | D | 50.92% |  | 439 | E | G | 13.65% |
|  | 542 | M | I | 27.45% |  | 284 | F | L | 18.87% |  | 442 | E | G | 27.76% |
|  | 556 | N | S | 99.39% |  | 290 | E | G | 99.69% | NS5B | 84 | S | P | 17.18% |
|  | 561 | L | V | 27.45% |  | 291 | E | G | 99.08% |  | 110 | N | S | 99.08% |
|  | 620 | M | I | 40.18% |  | 294 | R | G | 99.39% |  | 116 | V | L | 99.23% |
| NS4A | 2 | T | A | 99.54% |  | 296 | V | A | 99.39% |  | 147 | V | L | 68.71% |
|  | 29 | I | V | 99.54% |  | 299 | A | T | 98.62% |  | 261 | H | Y | 50.61% |
|  | 44 | L | R | 99.23% |  | 305 | R | K | 25.00% |  | 309 | Q | R | 22.85% |
| NS4B | 31 | Q | H | 49.54% |  | 350 | P | L | 95.86% |  | 311 | C | Y | 99.54% |
|  | 52 | K | R | 99.69% |  | 360 | T | A | 99.39% |  | 338 | V | A | 100.0% |
|  | 75 | V | A | 76.69% |  | 378 | K | E | 98.77% |  | 411 | Y | N | 50.77% |
|  | 216 | N | S | 10.74% |  | 380 | F | S | 98.93% |  | 547 | L | M | 16.72% |
|  | 240 | R | L | 27.30% |  | 384 | G | E | 99.08% |  | 549 | G | S | 71.47% |
|  | 242 | I | M | 99.54% |  | 390 | S | R | 98.16% |  | 581 | V | A | 99.39% |
|  | 255 | E | A | 90.64% |  | 392 | T | M | 88.96% |  |  |  |  |  |

**Supplementary Table 2**

**Oligonucleotides used as PCR primers**

| Primers | Sequence | Base position* | Purpose |
| --- | --- | --- | --- |
| Fw1 | TGTTTAGTCGAGGTTAAAAAAACGTC | - | RT-PCR for NS3 region (replicon) |
| Fw2 | ACAGGTCGGGACAAGAACCAG | 3142-3162 | RT-PCR for NS3 region (non-LT) |
| Fw3 | ACAAGAACCAGGTCGATGGGGAG | 3152-3174 | nested PCR for NS3 region (non-LT) |
| Fw4 | GGAGATACTTCTAGGACCGGCCGAT | 3024-3048 | RT-PCR for NS3 region (LT) |
| Fw5 | GCCTATCACGGCCTATTCCCAACAA | 3081-3105 | nested PCR for NS3 region (LT) |
| Rv1 | CTATTGGCCTGGAGTGTTTAGCTC | 9039-9069 | RT-PCR for NS5B region (replicon Year 0) |
| Rv2 | TTGGCCTGGAGTGTTTAGCTC | 9039-9066 | RT-PCR for NS5B region (replicon Year10 (Line1)) |
| Rv3 | TTGGCCTGGAGTGTGTAACTC | 9039-9066 | RT-PCR for NS5B region (replicon Year10 (Line2)) |
| Rv3 | CCCGTCACGTAGTGGAAATC | 6311-6292 | RT-PCR for NS5A region (non-LT) |
| Rv4 | GCGTAACCTCCACGTACTCC | 6283-6264 | nested PCR for NS5A region (non-LT) |
| Rv5 | CGTAGGAGAGGTTTGCACACTGGAG | 6401-6423 | RT-PCR for NS5A region (LT) |
| Rv6 | CATGGGCATTTCACGTTGTCAGTGG | 6319-6341 | nested PCR for NS5A region (LT) |

*: counted from the start codon of Core region. RT; reverse transcription, PCR; polymerase chain reaction

**Supplementary Table 3**

**Error rate of SMRT platform calculated from the control study with HCV-containing plasmid**

|  |  | average length | average coverage | matched  bases  (A) | unmatched bases  (B) | total error rate  (B/(A+B)) | mismatched bases  (C) | mismatched error rate (C/(A+B)) |
| --- | --- | --- | --- | --- | --- | --- | --- | --- |
| Plasmid 1 | pass1 | 2,717 bp | 1,067 | 5,595,147 bp | 221,706 bp | 3.811 % | 15,225 bp | 0.262 % |
|  | pass2 | 2,717 bp | 1,090 | 7,319,146 bp | 181,421 bp | 2.419 % | 8,776 bp | 0.117 % |
|  | pass3 | 2,563 bp | 2,298 | 8,895,652 bp | 166,942 bp | 1.842 % | 5,958 bp | 0.066 % |
|  | pass4 | 2,528 bp | 975 | 9,270,173 bp | 137,666 bp | 1.463 % | 4,504 bp | 0.048 % |
|  | pass5 | 2,579 bp | 7,201 | 9,432,568 bp | 97,535 bp | 1.023 % | 1,982 bp | 0.021 % |
|  | pass6 | 2,595 bp | 6,538 | 9,896,657 bp | 94,414 bp | 0.945 % | 2,165 bp | 0.022 % |
|  | pass7 | 2,589 bp | 6,508 | 11,056,355 bp | 59,053 bp | 0.531 % | 977 bp | 0.009 % |
|  | pass8 | 2,579 bp | 4,832 | 12,031,325 bp | 67,544 bp | 0.558 % | 842 bp | 0.007 % |
|  | pass9 | 2,527 bp | 3,010 | 12,610,798 bp | 64,552 bp | 0.509 % | 791 bp | 0.006 % |
|  | pass10 | 2,272 bp | 1,325 | 7,268,868 bp | 39,097 bp | 0.535 % | 470 bp | 0.006 % |
|  |  |  |  |  |  |  |  |  |
| Plasmid 2 | pass1 | 2,742 bp | 1,671 | 5,703,644 bp | 220,655 bp | 3.725 % | 17,192 bp | 0.290 % |
|  | pass2 | 2,736 bp | 2,483 | 7,250,951 bp | 168,218 bp | 2.267 % | 8,992 bp | 0.121 % |
|  | pass3 | 2,628 bp | 2,488 | 8,392,449 bp | 145,585 bp | 1.705 % | 6,940 bp | 0.081 % |
|  | pass4 | 2,607 bp | 2,401 | 8,721,130 bp | 114,463 bp | 1.295 % | 5,856 bp | 0.066 % |
|  | pass5 | 2,632 bp | 7,695 | 9,535,796 bp | 89,540 bp | 0.930 % | 4,310 bp | 0.045 % |
|  | pass6 | 2,635 bp | 6,378 | 10,562,596 bp | 75,691 bp | 0.711 % | 4,176 bp | 0.039 % |
|  | pass7 | 2,630 bp | 4,920 | 13,759,660 bp | 75,556 bp | 0.546 % | 4,976 bp | 0.036 % |
|  | pass8 | 2,584 bp | 3,023 | 14,746,876 bp | 71,147 bp | 0.480 % | 5,425 bp | 0.037 % |
|  | pass9 | 2,316 bp | 1,233 | 5,325,054 bp | 25,181 bp | 0.471 % | 1,920 bp | 0.036 % |
|  | pass10 | 1,681 bp | 320 | 1,872,163 bp | 8,574 bp | 0.456 % | 850 bp | 0.045 % |

SMRT, single molecular real-time; CCS, circular consensus sequencing

unmatched bases, mismatched bases + deletions + insertions
